# Data for the size of cholesterol-fat micelles as a function of bile salt concentration and the physico-chemical properties of six liquid experimental pine-derived phytosterol formulations in a cholesterol-containing artificial intestine fluid

**DOI:** 10.1016/j.dib.2016.12.027

**Published:** 2016-12-21

**Authors:** Jinsoo Yi, Tine A. Knudsen, Anne-Louise Nielsen, Lars Duelund, Morten Christensen, Pablo Hervella, David Needham, Ole G. Mouritsen

**Affiliations:** a MEMPHYS/SPSE, Department of Physics, Chemistry, and Pharmacy, University of Southern Denmark, Odense M DK-5230, Denmark; b DuPont Nutritional Biosciences ApS, Edwin Rahrs Vej 38, Brabrand DK-8220, Denmark; c Danish Technological Institute, Kongsvang Allé 29, Aarhus DK-8000, Denmark

## Abstract

The data in this paper are additional information to the research article entiltled “Inhibition of cholesterol transport in an intestine cell model by pine-derived phytosterols” (Yi et al.,2016) [1]. The data derived from the measurement on six liquid formulations of commercial pine-derived phytosterol (CPP) by dynamic light scattering. The data cover micelle size and the zeta-potential for formulations with cholesterol including monoglyceride, oleic acid, and bile salt. The data demonstrate the critical effect of the bile salt concentration on the size of cholesterol-digested fat micelles.

**Specifications Table**

**Table:** 

Subject area	*Physics, Chemistry*
More specific subject area	*Size of fat micelles with bile salt and physico-chemical properties of cholesterol-digested fat micelles with formulated liquid pine-derived phytosterols*
Type of data	*Table, figure*
How data was acquired	*All of the data in the table and the graph were obtained by dynamic light scattering (DelsaMax pro, Beckman-Coulter Indianapolis, USA)*
Data format	*Analyzed, Raw*
Experimental factors	*The diameter of particles (in nm) and the zeta-potential (in mV)*
Experimental features	*Physico-chemical properties of six experimental pine-derived phytosterol formulations were measured by dynamic light scattering.*
	*Cholesterol-containing micelle size was measured in three different bile salt concentrations by dynamic light scattering.*
Data source location	*Odense, Denmark*
Data accessibility	*Data is within this article*

**Value of the data**

• Physico-chemical properties of the CPP formulations are determined for additional information on a potential oral administration test.
• The particle size of liquid phytosterol formulations is different in different formulations, but the size did not affect the phytosterol absorption into intestine cells.
• The micelle size as a function of bile salt concentration is important for application of bile salt (hereby, sodium taurocholate, ST) on intestine cell models. The presented data demonstrate that the concentration of bile salt is the critical factor for regulating the size of fat micelles.

## 1 Data

**Fig. 1:**
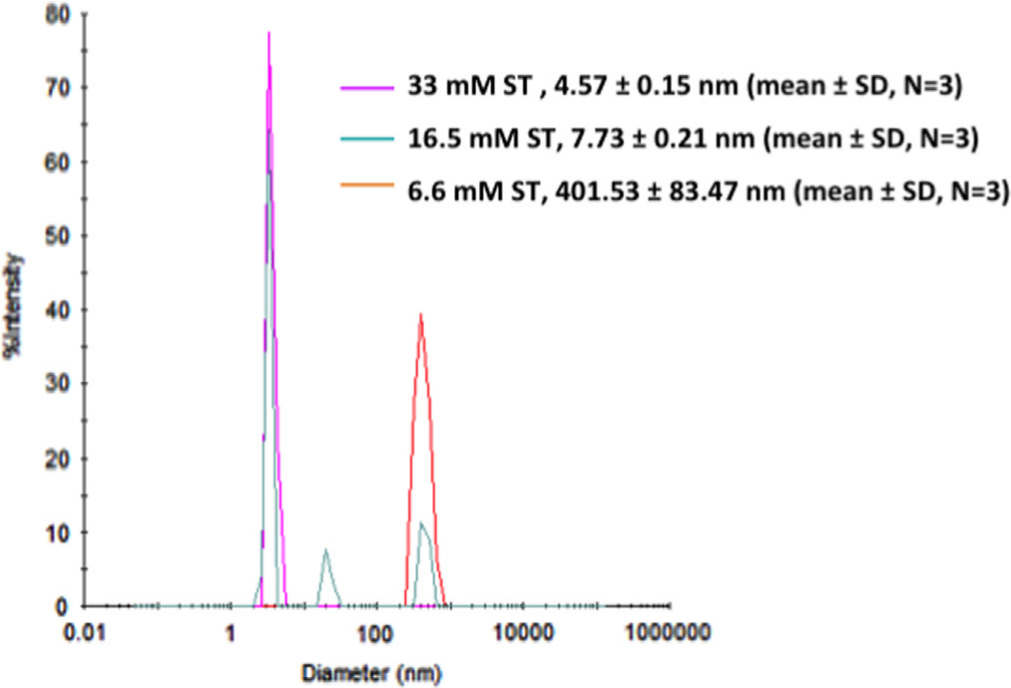
The size of lipophilic micelles, composed of monoglyceride, oleic acid, and cholesterol, as measured by a dynamic light scattering and as a function of the concentration of bile salt (sodium taurocholate, ST).

The data presented in this paper provides additional physico-chemical information on tested fat-micelles with six liquid formulations of commercial pine-derived phytosterol (CPP) and bile salt effect on cholesterol micelles size with monoglyceride (MG) and oleic acid (OA). Table 1 shows micelle size and zeta potential of CPP mixture with fat micelles. Fig. 1 shows the bile salt effect on the size of cholesterol micelles with MG and OA.

## 2 Experimental design, materials and methods

For the potential oral administration, the six experimental liquid formulations of commercial pine-derived phytosterol (CPP) reduced pH to 2 and increased pH again up to 7 to mimic the human digestive system (Yi et al., 2016) [1]. The concentration of β-sitosterol was different in the six experimental formulations and was supplied as 5% of each solution in water. To make 0.1 mM of β-sitosterol in working solution, 50.05 μl–74.11 μl of pH-adjusted six solutions was added in each of 0.1 mM of cholesterol, 0.5 mM of monoglyceride (MG), 1 mM of oleic acid (OA), 33 mM sodium taurocholate (ST) in Hanks buffered saline with magnesium and chloride (HBSS++). The particle size and the zeta-potential were measured by dynamic light scattering at 20 °C in triplicates (DelsaMax pro, Beckman-Coulter Indianapolis, USA).

To produce absorbable cholesterol-included fat micelles with micelle diameter of 3–25 nm (Ashworth and Lawrence, 1966) [2] for application to intestine cells, the bile salt concentration is critical in the intestine fluid. 0.1 mM of cholesterol, 0.5 mM of MG, and 1 mM of OA were mixed with 33 mM, 16.5 mM, and 6.6 mM of ST, and the size of cholesterol-containing micelles was measured by dynamic light scattering (DelsaMax pro, Beckman-Coulter Indianapolis, USA).

## Figures and Tables

**Table 1:** Sample preparation for the potential oral administration test of the commercial pine-derived phytosterol formulations with a cholesterol-containing artificial intestine fluid (Chol_AIF).

**CPP formulation**	**Chol_AIF+sample composition**	**Diameter (nm)**	**Zeta-potential (mV)**
	CPP_A (0,1 mM β-sitosterol)	220.8±251.76	−47.68±26.84
Formulation A	0.1 mM cholesterol		
	0.5 mM MG/1 mM OA		
	33 mM sodium taurocholate		
	0.01 M HBSS++		
	CPP_B (0,1 mM β-sitosterol)	590.6±78.32	−49.20±15.58
Formulation B	0.1 mM cholesterol		
	0.5 mM MG/1 mM OA		
	33 mM sodium taurocholate		
	0.01 M HBSS++		
	CPP_C (0,1 mM β-sitosterol)	432.23±18.52	−55.06±2.43
Formulation C	0.1 mM cholesterol		
	0.5 mM MG/1 mM OA		
	33 mM sodium taurocholate		
	0.01 M HBSS++		
	CPP_D (0,1 mM β-sitosterol)	50.8±49.61	−53.01±2.27
Formulation D	0.1 mM cholesterol		
	0.5 mM MG/1 mM OA		
	33 mM sodium taurocholate		
	0.01 M HBSS++		
	CPP_E (0,1 mM β-sitosterol)	23.4±4.14	−48.42±11.42
			
Formulation E	0.1 mM cholesterol		
	0.5 mM MG/1 mM OA		
	33 mM sodium taurocholate		
	0.01 M HBSS++		
	CPP_F (0,1 mM β-sitosterol)	459.83±67.42	−52.06±4.48
Formulation F	0.1 mM cholesterol		
	0.5 mM MG/1 mM OA		
	33 mM sodium taurocholate		
	0.01 M HBSS++		

PP: commercial pine-derived phytosterol product

The all values are mean±SE (*N*=3)
